# Supplementary material for: Chitosan–Cu Catalyzed Novel Ferrocenated Spiropyrrolidines: Green Synthesis, Single Crystal X-ray Diffraction, Hirshfeld Surface and Antibacterial Studies
Source: Polymers (Basel). 2023 Jan 13;15(2):429. doi: 10.3390/polym15020429 (PMC9867216; doi:10.3390/polym15020429)
Supplement: Supplementary file 1 [file polymers-15-00429-s001.zip › polymers-2077605-supplementary.pdf]

Chitosan−Cu Catalyzed Novel Ferrocenated Spiropyrrolidines: Green Synthesis, Single Crystal X-ray Diffraction, Hirshfeld Surface and Antibacterial Studies

Mohammad Asad [1,2,*], Muhammad Nadeem Arshad [1,2], Abdullah M. Asiri [1,2], Mohammed M. Rahman [1,2], Snigdha Kumaran [3] and Mohammed Musthafa Thorakkattil Neerankuzhiyil [3,*]

[1] Chemistry Department, Faculty of Science, King Abdulaziz University, P.O. Box 80203, Jeddauh 21589, Saudi Arabia
[2] Center of Excellence for Advanced Materials Research (CEAMR), King Abdulaziz University, P.O. Box 80203, Jeddah 21589, Saudi Arabia
[3] Research & Postgraduate Department of Chemistry, MES Kalladi College, Mannarkkad 678583, India
* Correspondence: magkhan@kau.edu.sa (M.A.); drmusthafa@meskc.ac.in (M.M.T.N.)

Table S1. Bond Lengths for **3a.**

Atom	Atom	Length/Å	Atom	Atom	Length/Å
C1	C2	1.530(4)	C19	C20	1.426(4)
C1	C4	1.567(4)	C19	C23	1.425(4)
C1	C12	1.515(4)	C19	Fe1	2.067(3)
C2	C3	1.540(4)	C20	C21	1.412(5)
C2	C19	1.509(4)	C20	Fe1	2.046(4)
C3	C29	1.526(4)	C21	C22	1.412(5)
C3	N1	1.469(4)	C21	Fe1	2.044(4)
C4	C5	1.533(4)	C22	C23	1.408(4)
C4	C6	1.511(4)	C22	Fe1	2.031(3)
C4	N1	1.463(4)	C23	Fe1	2.041(3)
C5	N2	1.340(4)	C24	C25	1.421(6)
C5	O2	1.228(4)	C24	C28	1.399(6)
C6	C7	1.385(4)	C24	Fe1	2.050(4)
C6	C11	1.365(5)	C25	C26	1.410(6)
C7	C8	1.372(4)	C25	Fe1	2.047(4)
C7	N2	1.406(4)	C26	C27	1.377(7)
C8	C9	1.370(5)	C26	Fe1	2.035(4)
C9	C10	1.374(6)	C27	C28	1.415(6)
C10	C11	1.385(5)	C27	Fe1	2.038(4)
C12	C13	1.505(4)	C28	Fe1	2.032(4)
C12	O1	1.209(3)	C29	C30	1.513(4)
C13	C14	1.384(4)	C30	C31	1.394(4)
C13	C18	1.390(4)	C30	C35	1.380(4)
C14	C15	1.386(4)	C31	C32	1.372(4)
C15	C16	1.377(4)	C32	C33	1.374(4)
C16	C17	1.362(5)	C33	C34	1.373(4)
C16	Br1	1.898(3)	C33	O3	1.382(4)
C17	C18	1.376(5)	C34	C35	1.389(5)

Table S2. Bond Angles for **3a.**

Atom	Atom	Atom	Angle/°	Atom	Atom	Atom	Angle/°
C4	C1	C2	104.3(2)	Fe1	C26	C25	70.2(2)
C12	C1	C2	115.7(2)	Fe1	C26	C27	70.3(3)
C12	C1	C4	112.5(2)	C28	C27	C26	108.1(4)
C3	C2	C1	102.1(2)	Fe1	C27	C26	70.2(3)
C19	C2	C1	112.2(2)	Fe1	C27	C28	69.4(2)
C19	C2	C3	115.0(2)	C27	C28	C24	108.2(4)
C29	C3	C2	117.0(3)	Fe1	C28	C24	70.7(2)
N1	C3	C2	105.4(2)	Fe1	C28	C27	69.9(2)
N1	C3	C29	109.6(2)	C30	C29	C3	111.6(3)
C5	C4	C1	108.4(2)	C31	C30	C29	119.9(3)
C6	C4	C1	114.9(2)	C35	C30	C29	123.6(3)
C6	C4	C5	102.1(2)	C35	C30	C31	116.5(3)
N1	C4	C1	104.0(2)	C32	C31	C30	122.3(3)
N1	C4	C5	112.3(2)	C33	C32	C31	119.8(3)
N1	C4	C6	115.2(3)	C34	C33	C32	119.8(3)
N2	C5	C4	108.1(3)	O3	C33	C32	117.1(3)
O2	C5	C4	124.5(3)	O3	C33	C34	123.1(3)
O2	C5	N2	127.4(3)	C35	C34	C33	119.8(3)
C7	C6	C4	108.2(3)	C34	C35	C30	121.9(3)
C11	C6	C4	131.9(3)	C4	N1	C3	111.3(2)
C11	C6	C7	119.8(3)	C7	N2	C5	111.9(3)
C8	C7	C6	122.0(3)	C20	Fe1	C19	40.58(11)
N2	C7	C6	109.4(3)	C21	Fe1	C19	68.17(13)
N2	C7	C8	128.6(3)	C21	Fe1	C20	40.39(13)
C9	C8	C7	117.5(3)	C22	Fe1	C19	68.60(13)
C10	C9	C8	121.4(4)	C22	Fe1	C20	68.31(15)
C11	C10	C9	120.5(4)	C22	Fe1	C21	40.54(14)
C10	C11	C6	118.7(3)	C23	Fe1	C19	40.59(12)
C13	C12	C1	119.5(3)	C23	Fe1	C20	67.88(14)
O1	C12	C1	120.4(3)	C23	Fe1	C21	67.71(14)
O1	C12	C13	120.2(2)	C23	Fe1	C22	40.46(12)
C14	C13	C12	123.4(3)	C24	Fe1	C19	110.99(14)
C18	C13	C12	118.3(3)	C24	Fe1	C20	127.38(16)
C18	C13	C14	118.3(3)	C24	Fe1	C21	162.25(19)
C15	C14	C13	121.4(3)	C24	Fe1	C22	156.99(19)
C16	C15	C14	118.3(3)	C24	Fe1	C23	124.05(16)
C17	C16	C15	121.6(3)	C25	Fe1	C19	124.37(16)
Br1	C16	C15	118.4(3)	C25	Fe1	C20	109.49(17)
Br1	C16	C17	120.0(2)	C25	Fe1	C21	124.12(18)
C18	C17	C16	119.7(3)	C25	Fe1	C22	158.87(17)
C17	C18	C13	120.8(3)	C25	Fe1	C23	159.94(17)
C20	C19	C2	127.0(3)	C25	Fe1	C24	40.57(17)
C23	C19	C2	126.6(3)	C26	Fe1	C19	158.4(2)
C23	C19	C20	106.3(3)	C26	Fe1	C20	121.63(19)
Fe1	C19	C2	129.6(2)	C26	Fe1	C21	106.05(18)
Fe1	C19	C20	68.93(18)	C26	Fe1	C22	121.44(18)
Fe1	C19	C23	68.72(18)	C26	Fe1	C23	158.44(19)
C21	C20	C19	108.5(3)	C26	Fe1	C24	67.83(19)
Fe1	C20	C19	70.49(19)	C26	Fe1	C25	40.40(17)
Fe1	C20	C21	69.7(2)	C27	Fe1	C19	161.8(2)

C22	C21	C20	108.3(3)	C27	Fe1	C20	154.69(19)
Fe1	C21	C20	69.89(19)	C27	Fe1	C21	118.84(18)
Fe1	C21	C22	69.3(2)	C27	Fe1	C22	105.07(18)
C23	C22	C21	107.6(3)	C27	Fe1	C23	123.8(2)
Fe1	C22	C21	70.2(2)	C27	Fe1	C24	67.81(18)
Fe1	C22	C23	70.12(18)	C27	Fe1	C25	67.4(2)
C22	C23	C19	109.2(3)	C27	Fe1	C26	39.51(19)
Fe1	C23	C19	70.69(17)	C28	Fe1	C19	126.55(17)
Fe1	C23	C22	69.42(19)	C28	Fe1	C20	163.66(18)
C28	C24	C25	107.4(4)	C28	Fe1	C21	154.8(2)
Fe1	C24	C25	69.6(2)	C28	Fe1	C22	120.37(19)
Fe1	C24	C28	69.2(2)	C28	Fe1	C23	108.83(17)
C26	C25	C24	107.3(5)	C28	Fe1	C24	40.09(18)
Fe1	C25	C24	69.8(2)	C28	Fe1	C25	67.7(2)
Fe1	C25	C26	69.4(2)	C28	Fe1	C26	67.5(2)
C27	C26	C25	108.9(4)	C28	Fe1	C27	40.71(18)

Table S3. Torsion Angles for **3a**.

A	B	C	D	Angle/°	A	B	C	D	Angle/°
C1	C2	C3	C29	-154.0(2)	C7	C8	C9	C10	-2.2(4)
C1	C2	C3	N1	-32.0(2)	C8	C9	C10	C11	1.9(5)
C1	C2	C19	C20	-153.6(2)	C12	C13	C14	C15	-177.5(3)
C1	C2	C19	C23	30.2(3)	C12	C13	C18	C17	178.0(3)
C1	C2	C19	Fe1	-61.4(2)	C13	C14	C15	C16	-0.8(3)
C1	C4	C5	N2	-116.0(2)	C13	C18	C17	C16	0.0(4)
C1	C4	C5	O2	61.3(3)	C14	C15	C16	C17	-0.0(4)
C1	C4	C6	C7	111.4(3)	C14	C15	C16	Br1	-179.3(2)
C1	C4	C6	C11	-66.4(3)	C15	C16	C17	C18	0.4(4)
C1	C4	N1	C3	6.2(3)	C19	C20	C21	C22	-1.3(3)
C1	C12	C13	C14	-9.2(3)	C19	C20	C21	Fe1	-60.0(3)
C1	C12	C13	C18	172.0(3)	C19	C23	C22	C21	-0.8(3)
C2	C3	C29	C30	-158.8(3)	C19	C23	C22	Fe1	59.7(3)
C2	C3	N1	C4	16.3(3)	C20	C21	C22	C23	1.3(3)
C2	C19	C20	C21	-176.0(3)	C20	C21	C22	Fe1	-59.1(3)
C2	C19	C20	Fe1	124.4(3)	C21	C22	C23	Fe1	-60.5(3)
C2	C19	C23	C22	176.9(3)	C24	C25	C26	C27	-0.1(4)
C2	C19	C23	Fe1	-124.3(3)	C24	C25	C26	Fe1	59.8(3)
C3	C29	C30	C31	73.0(3)	C24	C28	C27	C26	-0.8(4)
C3	C29	C30	C35	-104.7(3)	C24	C28	C27	Fe1	-60.5(4)
C3	N1	C4	C5	123.3(3)	C25	C26	C27	C28	0.6(4)
C3	N1	C4	C6	-120.4(3)	C25	C26	C27	Fe1	59.9(3)
C4	C5	N2	C7	-3.7(3)	C26	C27	C28	Fe1	59.8(4)
C4	C6	C7	C8	-176.0(2)	C29	C30	C31	C32	-178.3(3)
C4	C6	C7	N2	4.0(2)	C29	C30	C35	C34	178.2(3)
C4	C6	C11	C10	175.2(4)	C30	C31	C32	C33	0.1(4)
C5	N2	C7	C6	-0.1(3)	C30	C35	C34	C33	-0.0(4)
C5	N2	C7	C8	179.8(3)	C31	C32	C33	C34	0.4(4)
C6	C7	C8	C9	0.2(4)	C31	C32	C33	O3	-179.2(3)
C6	C11	C10	C9	0.5(4)	C32	C33	C34	C35	-0.4(4)

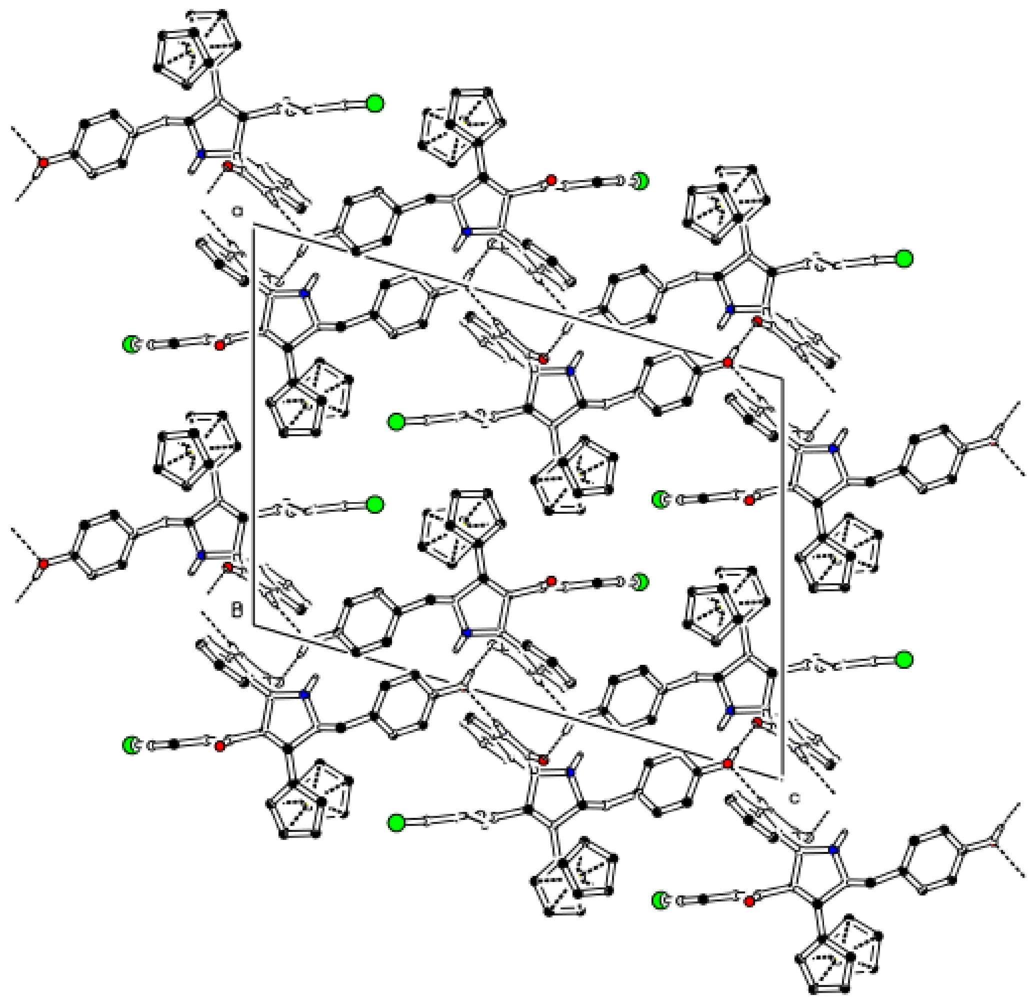

Figure S1. Showing the hydrogen bonding interaction and ring motif $R_4^4(12)$ formation, hydrogen atoms not involved in hydrogen bonding have been omitted for clarity.
